# Monthly Summary

**Published:** 1889-01

**Authors:** 


### Monthly Summary.

---

HOW SHALL THE SURGEON DISINFECT HIS INSTRUMENTS?—*By Dr. Hugo Davidsohn, (Berlin.)*—It has been demonstrated by bacteriological observations that wounds are less often infected by germs deposited by the air than by contact with the skin of the patient, the hands of the physician, instruments, sponges, dressings, etc. The author has endeavored to devise a method which shall insure the wound against infection from instruments, and at the same time be simple enough to find its place in the consulting room of the surgeon and the room of the patient. He found that disinfection with moist heat in a water bath, *i. e.*, boiling, is a reliable, perfect and practical method of disinfection. Care must be taken, however, that the heat penetrates to every place of the instrument, and that the air enclosed in the crevices of complicated instruments be totally expelled. Boiling for five minutes is usually sufficient, except in the case of instruments that are poor conductors, such as pessaries, hard rubber canulas, when a longer time is required. During the entire time of boiling the water bath should be kept at a uniform temperature of 100° C.; this may be secured if the cold air is prevented from entering the bath by covering the vessel and allowing the steam to escape through a small opening.

Immediately after the operation, the instruments are placed in cold water and all superficial pus, blood, or other matter brushed off. They are then boiled for five minutes in the water-bath, and after removal dried with a sterilized towel. Before the operation, they are again boiled for five minutes, allowed to cool, and used without addition of antiseptic solutions.

Although germs enclosed in pus and blood are not easily destroyed, since these substances are coagulated by high temperatures and then impede the passage of heat to the interior, the above described method insures a complete destruction of all pathogenic organisms.—Translanted from *Allgemeine medicinische Central-Zeitung, No.* 72, 1888.

---

TREATMENT OF CARBUNCLE.—*By Dr. R. F. Weir*—The author has treated five cases of carbuncle by scraping or erasion with prompt relief of the local and general symptoms.

An anæsthetic was used in four of the cases; in one cocaine was tried and succeeded tolerably well (though a great deal of difficulty was experienced in introducing the needle of the hypodermic syringe, on account of the tough character of the skin of the neck, which was rendered apparently much more resistant by the inflammatory process going on.) The slough was exposed either by the use of the scalpel or by Volkmann's sharp spoon tearing through the bridge of perforated skin until the yellow infiltrated tissues were reached. The affected parts were scraped out in a very elaborate manner, and their yellow prolongations, which were found in most of the cases to run up under the thick skin toward the skull, were followed up and scraped out with smaller spoons as far as possible. Whenever a mass of such character was found in the skin or among the muscles it was scraped out in like manner. The bleeding is apt to be quite profuse, though easily checked by pressure. After washing out the parts with a sublimate solution (1 to 1,000) the whole cavity, and especially those prolongations referred to above, are carefully packed with strips of a thirty per cent. iodoform gauze, and iodoform freely dusted over. A sublimate dressing is placed over the wound and secured with a four-tailed bandage, two ends of which are wound around the forehead, the other around the neck and under the arms to the back, to secure everything in proper position.—*The Medical Record*, July 14, 1888.

---

THE SPREAD OF ANTISEPSIS.—The advocates of antisepsis have good cause for hearty self and mutual congratulation, in looking over the vast results achieved in the last few years. Time was, and not so very long ago, when this great reform was wholly confined to its inventor and his immediate friends and adherents. From this numerically small beginning it has spread, by sheer force of merit, until there is not a civilized country which has not felt its beneficial influence. Against it have been arrayed sectional hate and professional prejudice. Against it stood every man who was wedded to any other system of treatment whatever—every man who was unwilling to acknowledge that his past surgical life had, to a certain extent, been a mistake. Not the least of its opponents has been that compound of dislike and novelty, respect for old traditions, intense prejudice and laziness, which is called "old fogyism."

Against these and other obstacles antisepsis has made its way—not by compulsion, but appealing rather to the conscience of the profession; only needing a fair trial to be appreciated; never losing a true convert, until the completeness of its conquest is astonishing even to enthusiasts.

The advantage of such a uniform practice as this, both to profession and people, is incalculable. To the former it is a surgical volapuk—a common standard by which one's own work and that of his brethren can be estimated and compared; to the latter it offers a reasonable assurance that they will receive the best of treatment, no matter into whose hands they may fall.

By no means the least gratifying feature of this reform is the stand taken by our brethren of the country and smaller towns. It was to be expected that hospital surgeons everywhere, and practitioners in larger cities, where the results of antiseptics were forced upon their attention, would fall promptly in to line if only as a matter of self protection. But the fact that men who are masters of the situation in their own localities, should voluntarily array themselves on the right side, is only another proof of their professional intelligence and conscientiousness.

It has lately been the duty of the writer to take surgical cognizance of some twenty cases injured in a railroad accident, They were treated by half a dozen physicians from three different towns. In not one of these cases was there any suppuration, nor any other ill result attributable to treatment.—*Dental Register.*

---

VIRCHOW ON ARTIFICIAL DEFORMITIES.—What is the use, says Virchow, of introducing the principles and appliances of hygiene into the huts of the poor and ignorant, when the scions of wealth, pretended intelligence, and fashion, especially in the gentler sex, show their contempt of hygiene by their dress and general wearing apparel. In days gone by, the speaker said, I have battled against the diabolical invention called the corset, but this crusade has been given up by me as absolutely futile. The French shoe, with pointed toe and the heel under the middle of the foot, is anothes fashion which, on account of its detrimental influences on the body, cannot be opposed too energetically. Even the modern stocking, pointed and symmetrical, is injurious to health, as it does not conform to the anatomical formation of the foot. To offset this we notice that the professor of pathology at the University of Cambridge, Dr. C. S. Roy, and his demonstrator, Mr. G. Adams, have appeared as champions of tight-lacing. Moderate pressure of the abdomen, they say, tends to assist the emptying of the abdominal veins, and experiment showed that the amount of blood expelled by the heart at each stroke was greater, and that consequently the blood-supply of the brain and spinal cord, the muscles and skin, was increased. This increased supply, they say, is useful when violent muscular exertions were being made; consequently tight lacing in women and tight waistbands in men are useful.—*Medical Record.*

---

DISINFECTION OF SURGICAL INSTRUMENTS.—*By Dr. Redard.*—The disinfection by heat is the best procedure; unfortunately every instrument cannot be passed through a flame. The immersion in liquids at a high temperature is only useful when the liquid is at least 110° C. and the contact very prolonged. The author's experiments show that catheters, trochars, mouse-tooth forceps are only sterilized by remaining at least three-quarters of an hour in a liquid of the temperature between 110° and 120° C: knives require only ten minutes. Disinfection by means of steam under pressure for fifteen or twenty minutes renders the instruments perfectly sterile.—Translated from *Revue de Chirurgie.*

---

OXYGEN NOT SO IMPORTANT IN RESPIRATION.—M. Brown-Sequard, who is nothing if not original, recently read a note at the Societe de Biologie in which he asserted that oxygen was not so necessary to respiration as had been supposed, and that carbonic-acid gas was not so poisonous as had been claimed. He had succeeded in keeping animals alive and comfortable in an atmosphere containing sixty per cent. of carbonic-aid gas, while he and M. D'Arsomal had been able to breathe an atmosphere containing twenty per cent. of CO2 without any inconvenience. It is usually taught that air containing ten per cent. of CO2 is poisonous. Brown-Sequard thinks that nitrogen is a very important element in respiration.—*Medical Record.*

---

VERDICT OF MALPRACTICE IN ADMINISTRAITON OF CHLOROFORM.—*The Lancet* states that a death from chloroform occurred in Sydney; and a lawsuit brought by the husband of the deceased resulted in a verdict of guilty, setting the damages at £200, on the ground that the anæsthetic was improperly administered.

BEEF, MILK AND PHTHISIS.—There can be no doubt that bovine and human tuberculosis are essentially the same disease, and that bovine tuberculosis may be transmitted to man by means of infected milk and meat. The necessity of prophylactic measures with this point in view has been urged by the "Record" for many years, and we are glad to see that the public press and legislatures have at last begun to recognize the fact. It is not probable, however, that the cow is the source of phthisical contagion in a large proportion of cases. The (pulmonary) phthisical age is from fifteen to thirty, after which milk ceases to be an important article of food. Further investigation, however, on this point is needed. —*Fx.*

---

OPIUM SMOKING.—The English colonial physician in Hong Kong refers in his last annual report to the opium-smoking habit among the chinese, and holds that it is hardly worse in its effects, when used in moderation, than tobacco smoking. The sudden abstraction of opium from those accustomed to smoke it causes no special physical disturbance. He has seen numbers of hearty old men who had smoked opium all their lives, yet were in good flesh and strength, enjoyed a fine appetite, and slept the sleep of the just. He maintains that the injurious effects of this habit are not to be compared with those of the abuse of alcohol.—*Ex.*

---

EXTIRPATION OF THE LARYNX.—Dr. Caselli reported a case of a young girl, aged 19, who had suffered for a year from difficulty in swallowing, and paroxysms of cough and dyspepsia which were at times so violent as to threaten life. An extensive epitheliomatous infiltration of the pharynx and larynx was found. The author removed the larynx, the pharynx, the base of the tongue, the two tonsils and all the soft parts of the palate. Recovery was complete one month after the operation with relief of all the symptoms.—Translated from *Gazette Med. de Paris*, 1888

